# First results of the “Lean European Open Survey on SARS-CoV-2-Infected Patients (LEOSS)”

**DOI:** 10.1007/s15010-020-01499-0

**Published:** 2020-10-01

**Authors:** Carolin E. M. Jakob, Stefan Borgmann, Fazilet Duygu, Uta Behrends, Martin Hower, Uta Merle, Anette Friedrichs, Lukas Tometten, Frank Hanses, Norma Jung, Siegbert Rieg, Kai Wille, Beate Grüner, Hartwig Klinker, Nicole Gersbacher-Runge, Kerstin Hellwig, Lukas Eberwein, Sebastian Dolff, Dominic Rauschning, Michael von Bergwelt-Baildon, Julia Lanznaster, Richard Strauß, Janina Trauth, Katja de With, Maria Ruethrich, Catherina Lueck, Jacob Nattermann, Lene Tscharntke, Lisa Pilgram, Sandra Fuhrmann, Annika Classen, Melanie Stecher, Maximilian Schons, Christoph Spinner, Jörg Janne Vehreschild

**Affiliations:** 1 Department I for Internal Medicine, University Hospital of Cologne, University of Cologne, Cologne, Germany; 2 German Center for Infection Research (DZIF), Brunswick, Germany; 3 Department of Infectious Diseases and Infection Control, Ingolstadt Hospital, Ingolstadt, Germany; 4 Department of Internal Medicine, Infectious Diseases, Goethe University Frankfurt, Frankfurt, Germany; 5 Technical University Munich, Children’s Hospital, Munich, Germany; 6 Research Unit Gene Vectors, Helmholtz Zentrum Muenchen, Munich, Germany; 7 Clinic for Pneumology, Infectiology, Internal Medicine and Intensive Care, Hospital Dortmund gGmbH, Dortmund, Germany; 8 Department of Gastroenterology and Infectiology, Heidelberg University Hospital, Heidelberg, Germany; 9 Department of Internal Medicine, University Hospital Schleswig-Holstein, Kiel, Germany; 10 Department of Gastroenterology and Infectiology, Klinikum Ernst-von-Bergmann, Potsdam, Germany; 11 Emergency Department, University Hospital Regensburg, Regensburg, Germany; 12 Department of Medicine II, University of Freiburg, Freiburg, Germany; 13 University Clinic for Haematology, Oncology, Haemostaseology and Palliative Care, University of Bochum, Minden, Germany; 14 Division of Infectious Diseases, Department of Internal Medicine III, University Hospital of Ulm, Ulm, Germany; 15 Division of Infectious Diseases, Department of Medicine II, University of Würzburg Medical Center, Würzburg, Germany; 16 Center of Infectiology Berlin/Prenzlauer Berg, Berlin, Germany; 17 Department of Neurology, Katholisches Klinikum Bochum, Ruhr University Bochum, Bochum, Germany; 18 4th Department of Internal Medicine, Klinikum Leverkusen gGmbH, Leverkusen, Germany; 19 Department of Infectious Diseases, University Hospital Essen, University Duisburg-Essen, Essen, Germany; 20 Bundeswehr Central Hospital, Koblenz, Germany; 21 Department of Internal Medicine III, Ludwig Maximilians University, Munich, Germany; 22 German Cancer Consortium (DKTK), partner site Munich, Munich, Germany; 23 Bavarian Center for Cancer Research (BZKF), Munich, Germany; 24 2nd Medical Clinic, Hospital Passau, Passau, Germany; 25 Department of Medicine 1, University Hospital Erlangen, Erlangen, Germany; 26 Medical Clinic II, University Hospital Gießen, Giessen, Germany; 27 Division of Infectious Diseases, University Hospital Carl Gustav Carus, Technical University of Dresden, Dresden, Germany; 28 Department of Internal Medicine II, University Hospital Jena, Jena, Germany; 29 Department of Haematology, Haemostasis, Oncology and Stem Cell Transplantation, Hannover Medical School, Hannover, Germany; 30 Department of Internal Medicine I, UKB University Hospital Bonn, Bonn, Germany; 31 Department of Internal Medicine II, School of Medicine, Technical University of Munich, University Hospital Rechts Der Isar, Munich, Germany; 32 Department of Internal Medicine, Hematology and Oncology, Goethe University Frankfurt, Frankfurt, Germany

**Keywords:** SARS-CoV-2, COVID-19, LEOSS, Cohort study

## Abstract

**Purpose:**

Knowledge regarding patients’ clinical condition at severe acute respiratory syndrome coronavirus 2 (SARS-CoV-2) detection is sparse. Data in the international, multicenter Lean European Open Survey on SARS-CoV-2-Infected Patients (LEOSS) cohort study may enhance the understanding of COVID-19.

**Methods:**

Sociodemographic and clinical characteristics of SARS-CoV-2-infected patients, enrolled in the LEOSS cohort study between March 16, 2020, and May 14, 2020, were analyzed. Associations between baseline characteristics and clinical stages at diagnosis (uncomplicated vs. complicated) were assessed using logistic regression models.

**Results:**

We included 2155 patients, 59.7% (1,287/2,155) were male; the most common age category was 66–85 years (39.6%; 500/2,155). The primary COVID-19 diagnosis was made in 35.0% (755/2,155) during complicated clinical stages. A significant univariate association between age; sex; body mass index; smoking; diabetes; cardiovascular, pulmonary, neurological, and kidney diseases; ACE inhibitor therapy; statin intake and an increased risk for complicated clinical stages of COVID-19 at diagnosis was found. Multivariable analysis revealed that advanced age [46–65 years: adjusted odds ratio (aOR): 1.73, 95% CI 1.25–2.42, *p* = 0.001; 66–85 years: aOR 1.93, 95% CI 1.36–2.74, *p* < 0.001; > 85 years: aOR 2.38, 95% CI 1.49–3.81, *p* < 0.001 vs. individuals aged 26–45 years], male sex (aOR 1.23, 95% CI 1.01–1.50, *p* = 0.040), cardiovascular disease (aOR 1.37, 95% CI 1.09–1.72, *p* = 0.007), and diabetes (aOR 1.33, 95% CI 1.04–1.69, *p* = 0.023) were associated with complicated stages of COVID-19 at diagnosis.

**Conclusion:**

The LEOSS cohort identified age, cardiovascular disease, diabetes and male sex as risk factors for complicated disease stages at SARS-CoV-2 diagnosis, thus confirming previous data. Further data regarding outcomes of the natural course of COVID-19 and the influence of treatment are required.

**Electronic supplementary material:**

The online version of this article (10.1007/s15010-020-01499-0) contains supplementary material, which is available to authorized users.

## Introduction

Since December 2019, the severe acute respiratory syndrome coronavirus 2 (SARS-CoV-2) originating in the Chinese city of Wuhan, located in Hubei Province, has spread globally and rapidly developed into a global pandemic [1, 2].

A comprehensive descriptive analysis of Chinese patients suffering from coronavirus disease (COVID-19) was published after processing the demographic data of 72,314 cases recorded by the Chinese Centers for Disease Control and Prevention [3]. The published study, in addition to other studies, identified risk factors for an advanced course of disease. The risk factors identified were age, male gender, higher body mass index (BMI), diabetes mellitus, and cardiovascular disease. Laboratory parameters such as elevated cytokine levels and altered coagulation parameters were discussed as indicators of possible hospitalization or disease severity. These parameters have been described extensively [4–12]. Many cohort studies have presented results about associating factors using data of single hospitals [9, 13], collaborations within a city [14] or a country [8, 11, 15–17], or transnational collaborations [10, 18, 19]. The population of studies is heterogeneous and only few data on large European transregional cohorts of SARS-CoV-2-infected patients exist, so far.

These publications provide important insights to improve the clinical management of the patients, but more comprehensive, large, transregional cohort data are urgently needed to identify the clinical characteristics of the infected patients. Information on possible epidemiological or clinical risk factors is still scarce.

Using an extensive database on the clinical course of disease, data from the international Lean European Open Survey on SARS-Infected Patients (LEOSS) cohort may improve understanding of the heterogeneous clinical course and risk factors of SARS-CoV-2-infected patients and effective management strategies to treat this infection. This analysis focuses on baseline characteristics and the clinical disease stage of the patients at the time of COVID-19 diagnosis.

## Methods

### Study design and patient cohort

**Fig. 1:**
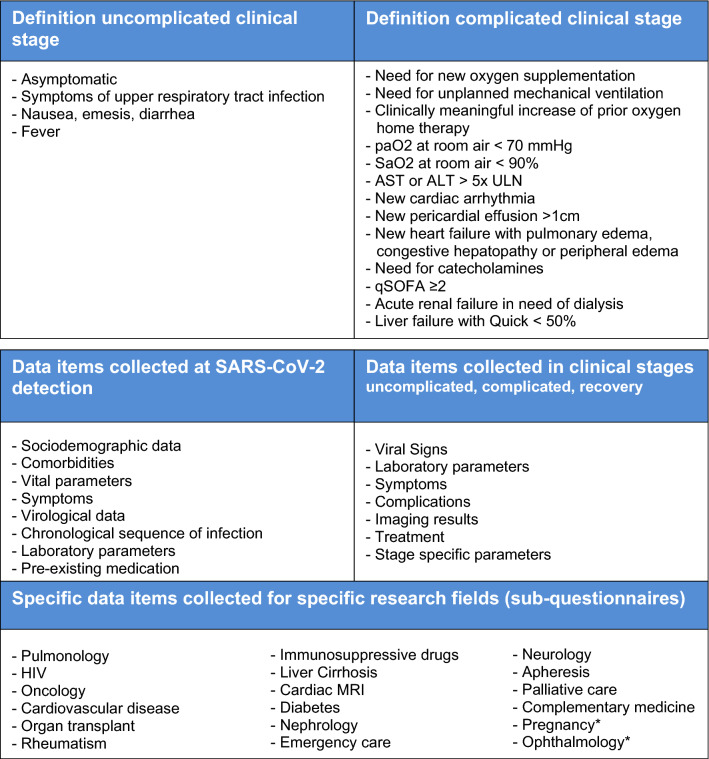
Top—definition of uncomplicated and complicated clinical stage. The respective stage is considered to be present if one of the criteria is met. Bottom—parameters included into Lean European Open Survey on SARS-CoV-2-Infected Patients (LEOSS). Sub-questionnaires under development at the time of submission of this manuscript are defined with asterisk (*). *paO2* partial pressure of oxygen, *SaO2* oxygen saturation, *AST* aspartate aminotransferase, *ALT* alanine transaminase, *ULN* upper limit of normal in the respective local laboratory, *qSOFA* quick sequential [sepsis-related] organ failure assessment

This analysis includes data of patients with confirmed SARS-CoV-2 infection (positive reverse transcriptase polymerase chain reaction (PCR) results), who received care at a LEOSS partner site (as inpatient or outpatient) between March 16, 2020, and May 14, 2020. Only patients with information available on follow-up and at the end of the treatment (recovery or death) were included in the analysis. An overview of recorded data in LEOSS is displayed in Fig. 1.

### Clinical data and endpoint

In our analysis, we considered baseline data available closest to the time of first positive SARS-CoV-2 test within 48 h. We examined patient characteristics (age, gender, BMI, smoking status, comorbidities, pre-existing Angiotensin-converting-enzyme inhibitors (ACE inhibitors), angiotensin II type 1 (AT1) receptor antagonists, statins, ibuprofen, and immunosuppressants intake, such as drugs for treatment of cancer, rheumatologic/inflammatory diseases, chronic inflammatory bowel disease, multiple sclerosis, etc.), vital parameters (body temperature, pulse, respiratory rate, oxygen saturation, oxygen, and carbon dioxide partial pressure), and laboratory parameters of acute phase and organ functions as well as soluble inflammation parameters. The primary endpoint was defined as a complicated clinical stage at initial presentation (time of first SARS-CoV-2 PCR-positive test result). A complicated clinical stage was defined by clinical findings (see Fig. 1) that indicate a clinical stage that requires medical treatment.

Parameters were documented categorically. Comorbidities were additionally dichotomized as cardiovascular disease (myocardial infarction, aortic stenosis, atrioventricular (AV) block, carotid arterial disease, chronic heart and circulation failure, peripheral vascular disease, hypertension, atrial fibrillation, coronary artery disease), pulmonary disease (chronic obstructive pulmonary disease, asthma, other chronic pulmonary diseases), hematological/oncological disease (leukemia, lymphoma, solid tumor, stem cell transplantation), diabetes mellitus (with and without end organ damage), kidney disease (acute kidney injury at time of SARS-CoV-2 detection, chronic kidney disease), neurological diseases (hemiplegia, dementia, cerebrovascular disease, stroke, transient ischemic attack, motoneuron diseases, movement disorder, multiple sclerosis, myasthenia gravis, neuromyelitis optica spectrum disorder, other neurological autoimmune diseases, other prior neurological diagnosis), others (connective tissue disease, peptic ulcer disease, chronic liver disease, liver cirrhosis, organ transplantation, rheumatic disease, HIV/AIDS). A comorbidity was defined as present if a minimum of one specific comorbidity was documented. Comorbidities were set to unknown/missing when all specific comorbidities of one group were unknown or missing. Values documented as unknown were defined as missing.

### Ethical statement

Data were recorded completely anonymous and no patient-identifying data were stored. Written patient informed consent was waived. Metric parameters were categorized, and granular time-varying data (not included in this study) were aggregated over the duration of specific COVID-19 stages. Approval for LEOSS was obtained by the applicable local ethics committees of all participating centers and registered at the German Clinical Trails Register (DRKS, No. S00021145).

### Statistical analysis

We calculated and reported patient characteristics as absolute numbers and percentages. Associations between clinical baseline characteristics and a complicated clinical stage at time of first positive SARS-CoV-2 detection (dependent variable) were assessed using a univariate and a multivariable logistic regression model. The presence of multicollinearity problems was assessed among the explanatory variables using the tolerance and Variance Inflation Factor (VIF). Additional parameters considered for model section include AIC (Akaike information criterion) and BIC (Bayesian information criterion). See Supplement Table 2 for further sensitivity analysis. Odds ratios (ORs) with 95% confidence intervals (CIs) were calculated to assess the strength of the association. The level of significance was chosen to be *p* < 0.05.

### Data collection and processing

Data were recorded in an electronic case report form (eCRF) operated using the online cohort platform ClinicalSurveys.net, which was developed by the University Hospital of Cologne (UHC). ClinicalSurveys.net was hosted by QuestBack, Oslo, Norway on servers of UHC, Cologne, Germany, as part of a software-as-a-service agreement. Data were processed on the servers of UHC. Data management, statistical analysis, and computation of figures were conducted using R (R Development Core Team, Vienna, Austria, Version 3.5.2., 2019). Additional information about the LEOSS questionnaire can be found under https://leoss.net/.

## Results

### Cohort population

**Fig. 2:**
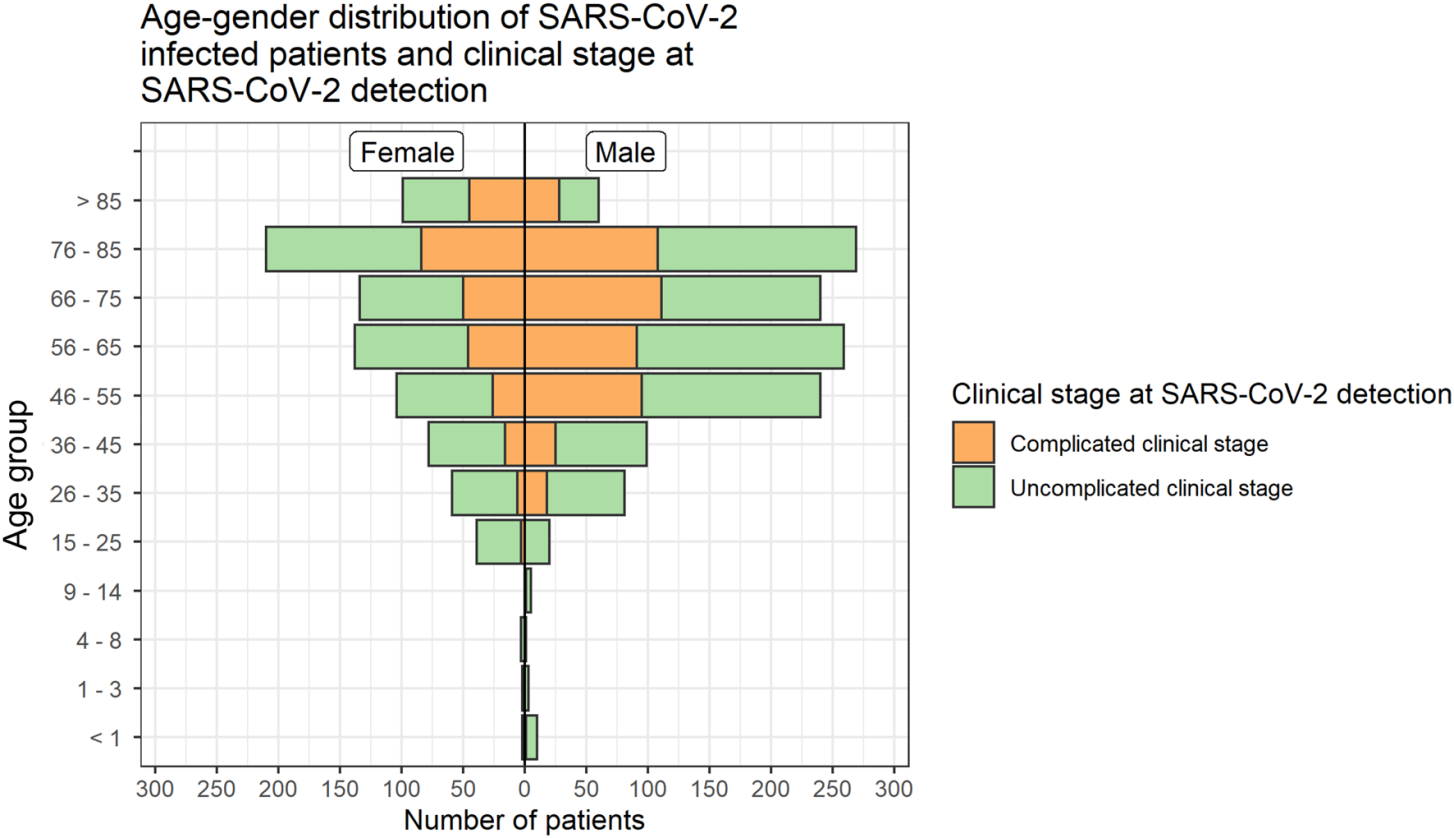
Age–gender distribution and clinical stage at time of SARS-CoV-2 detection. The bars are divided according to the clinical condition at time of SARS-CoV-2 detection (uncomplicated vs. complicated). The number of individuals in the total population is 2155

We included 2155 patients from university hospitals (58.2%; 1254/2155), non-university hospitals (36.4%; 784/2155), and general practitioners (5.4%; 117/2155). Data were collected at 112 European study sites, mainly from Germany (98.1%; 2113/2155), and Austria, Belgium, Italy, Switzerland, Latvia, Spain, Bosnia and Herzegovina, as well as Turkey. The median number of documented patients per study site was 5, with a minimum of 1 and a maximum of 162 documented patients. Most patients were Caucasian (95.6%; 1738/1818) and had an inpatient stay (92.8%; 1829/1971). Almost one-tenth of patients were documented as having an asymptomatic SARS-CoV-2 infection (9.9%; 161/1629). Patient characteristics are summarized in Table 1. Asymptomatic patients compared to all patients had more often documented comorbidities (see Supplements Table 1). This could be the reason that they were monitored without symptoms. The median estimated duration from SARS-CoV-2 infection to the day of the first SARS-CoV-2 PCR-positive test result was seven days (interquartile range; IQR: 5–11 days). Females were predominant in the age category 15–25 years (66.1%; 39/59) and > 85 years (62.3%; 99/159). Males were most predominant in the age category 46–65 years (67.3%; 499/741). The age–gender distribution is displayed in Fig. 2.

**Table 1:** Baseline characteristics of patients in an uncomplicated or complicated stage of disease at day of positive SARS-CoV-2 detection

	Total	Uncomplicated clinical stage at day of positive SARS-CoV-2 detection	Complicated clinical stage at day of positive SARS-CoV-2 detection
Included cases	2155	65% (1400/2155)	35% (755/2155)
Age (years)			
≤ 14	1.2% (26/2155)	1.6% (23/1400)	0.4% (3/755)
15–25	2.7% (59/2155)	4.0% (56/1400)	0.4% (3/755)
26–45	14.7% (317/2155)	18.0% (252/1400)	8.6% (65/755)
46–65	34.4% (741/2155)	34.5% (483/1400)	34.2% (258/755)
66–85	39.6% (500/2155)	35.7% (500/1400)	46.8% (353/755)
> 85	7.4% (159/2155)	6.1% (86/1400)	9.7% (73/755)
Sex			
Female	40.3% (868/2155)	42.2% (591/1182)	36.7% (277/554)
Male	59.7% (1287/2155)	57.8% (809/1400)	63.3% (478/956)
Body mass index (kg/m^2^)			
< 18.5	3.0% (36/1187)	3.3% (24/724)	2.6% (12/463)
18.5–24.9	36.6% (434/1187)	39.5% (286/724)	32.0% (148/463)
25–29.9	36.1% (428/1187)	36.2% (262/724)	35.9% (166/463)
30–34.9	15.7% (186/1187)	14.4% (104/724)	17.7% (82/463)
≥ 35	8.7% (103/1187)	6.6% (48/724)	11.9% (55/463)
Smoking			
Active smoking	13.8% (143/1040)	12.6% (92/731)	16.5% (51/309)
Former smoking	15.1% (157/1040)	13.5% (99/731)	18.8% (58/309)
Nonsmoking	71.2% (740/1040)	73.9% (540/731)	64.7% (200/309)
Comorbidities			
Cardiovascular disease	55.7% (1158/2079)	49.9% (678/1358)	66.6% (480/721)
Diabetes mellitus	18.4% (385/2090)	15.4% (210/1367)	24.2% (175/723)
Pulmonary disease	14.9% (307/2059)	13.4% (179/1336)	17.7% (128/723)
Hematological and/or oncological disease	14.6% (305/2087)	14.6% (200/1367)	14.6% (105/720)
Neurological disease	21.5% (411/1911)	19.5% (245/1257)	25.4% (166/654)
Kidney disease	14.6% (305/2088)	12.7% (173/1366)	18.3% (132/722)
Other comorbidities*	12.0% (243/2021)	12.4% (164/1319)	11.3% (79/702)
Pre-existing medication at day of positive SARS-CoV-2 test			
ACE inhibitors	18.1% (364/2008)	16.2% (216/1332)	21.9% (148/676)
AT-1-receptor antagonists	17.2% (345/2000)	16.3% (217/1330)	19.1% (128/670)
Statins	23.2% (335/1441)	21.1% (200/946)	27.3% (135/495)
Ibuprofen	4.1% (78/1918)	4.7% (60/1274)	2.8% (18/644)
Pre-existing medication until three months before positive SARS-CoV-2 test			
Any immunosuppressive medication	10.3% (189/1841)	11.0% (132/1202)	8.9% (57/639)

*ACE inhibitors* angiotensin-converting-enzyme inhibitor, *AT-1-receptor antagonists* angiotensin II type 1 (AT1) receptor antagonists. Other comorbidities—connective tissue disease, peptic ulcer disease, chronic liver disease, liver cirrhosis, organ transplantation, rheumatic disease, HIV/AIDS

### Vital and laboratory parameters

In 61.1% (1002/1641) of patients, the body temperature was below 38.0 °C within 48 h subsequent to the day of SARS-CoV-2 detection (could be in- or outpatient). The respiratory rate exceeded 21 breaths per minute in 32.1% (378/1178) of available cases on the day of SARS-CoV-2 detection. Oxygen saturation was below 90% in 16.9% (2268/1584) and the pulse rate exceeded 120 beats per minute in 4.8% (77/1603) of patients on the day of SARS-CoV-2 detection.

**Fig. 3:**
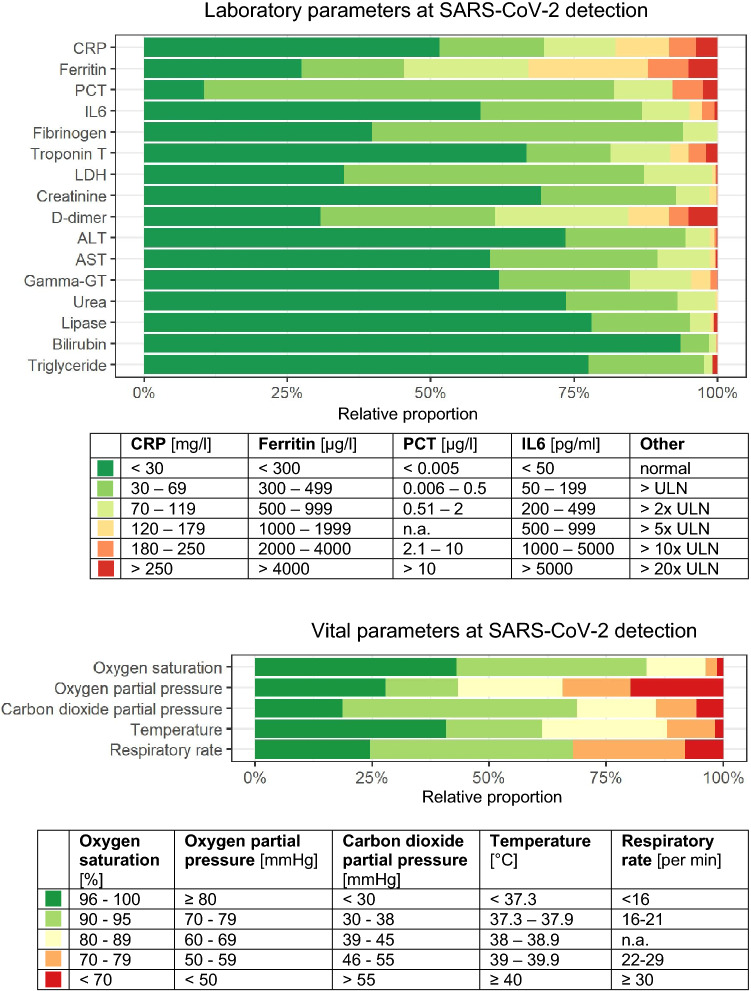
Clinical–chemical laboratory parameters and vital parameters at the time of SARS-CoV-2 detection. Relative proportions to the measured/collected parameters are shown (missing values were excluded). The colors (green to red) express an increase in impairment from the normal to slightly impaired range (green) to critically impaired (red). Normal values are CRP < 5 mg/l; ferritin male < 300, females < 150 µg/l; PCT < 0.5 ng/l; IL6 < 10 pg/ml; oxygen saturation 96–100%; oxygen partial pressure ≥ 80 mmHg; carbon dioxide partial pressure 35–45 mmHg; temperature < 37.3 °C; respiratory rate < 16 per min. *CRP* C-reactive protein, *PCT* procalcitonin, *IL6* interleukin-6, *LDH* lactate dehydrogenase, *ALT* alanine aminotransferase, *AST* aspartate aminotransferase, *Gamma-GT* gamma-glutamyl transferase, *ULN* upper limit of normal in the respective local laboratory. The number of individuals in the total population is 2155

Figure 3 shows the vital and laboratory parameters at the time of SARS-CoV-2 detection. Serum ferritin levels were above 300 µg/L in 72.5% (464/640) of patients with available values, C-reactive protein (CRP) was above 29 mg/L in 48.5% (759/1566), procalcitonin (PCT) was above 0.005 µg/L in 89.5% (716/800), interleukin-6 (IL6) was above 49 pg/mL in 41.3% (238/576). Fibrinogen was elevated in 60.2% patients (241/400), lactate dehydrogenase (LDH) in 65.1% (915/1405), as well as d-dimer in 38.8% (334/861) and was above the normal range of the respective local laboratory. The percentage of patients diagnosed in a complicated clinical stage of the disease and CRP values below 30 mg/L was 36.3% (222/612).

Possible surrogate markers of COVID-19 hepatopathy were higher in patients with SARS-CoV-2 infection than the age-adjusted normal value of the respective local laboratory: Aspartate aminotransferase (AST) in 39.6% (544/1372), alanine aminotransferase (ALT) in 26.6% (371/1396), and gamma-GT in 38.0% (502/1318). The hematologic parameters at the time of SARS-CoV-2 detection were as follows: leukocytes: < 3999 cells/µL in 17.2% (270/1573), 4000–11999/µL in 73.2% (1152/1573) ≥ 12,000/µL in 9.6% (151/1573) of patients; lymphocytes: < 799 cells/µL in 39.9% (487/1221) and ≥ 800 cells/µL in 61.2% (747/1221) of patients. Platelets: < 199,999 cells/µL in 11.9% (186/1559), 120,000–449,999 cells/µL in 84.9% (1323/1559), and ≥ 450,000 cells/µL in 2.2% (43/1559) of patients.

### Characteristics for clinical stage at SARS-CoV-2 detection

At the time of SARS-CoV-2 detection, 35.0% (755/2155) of patients, of whom 0.8% (6/755) were younger than 25 years, were already in a complicated clinical stage. Of those, the majority were male (63.3%; 478/755) and between 66 and 85 years of age (46.8%; 353/755). A total of 11.9% (55/463) and 16.5% (51/309) of patients in the complicated clinical stage at SARS-CoV-2 detection had a BMI of > 35 kg/m^2^ and documented active smoking, respectively. All documented comorbidities and all pre-existing medications, except underlying hematologic and/or oncologic disease, ibuprofen, and any immunosuppressants were more often reported for patients with a complicated rather than an uncomplicated clinical stage of disease at SARS-CoV-2 detection (Table 1). The proportion of documented intake of ACE inhibitors of patients with cardiovascular diseases was for patients in an uncomplicated or complicated clinical stage at SARS-CoV-2 detection 31.4% (uncomplicated: 206/656; complicated: 137/436) and considering only patients aged > 45 years, 32.1% (201/626) and 31.4% 135/430), respectively.

Univariate regression results are shown in Table 2. The multivariable regression model was adjusted for age, gender, underlying cardiovascular diseases, diabetes mellitus, and pulmonal diseases (Table 2). We identified that advanced age, compared to individuals between 26 and 45 years of age, was significantly associated with a higher risk for a complicated clinical stage at SARS-CoV-2 detection (46–65 years: adjusted OR; aOR 1.73, 95% CI 1.25–2.42, *p* = 0.001; 66–85 years: aOR 1.93, 95% CI 1.36–2.74, *p* ≤ 0.001; > 85 years: aOR 2.38, 95% CI 1.49–3.81, *p* ≤ 0.001, 15–25 years: aOR 0.23, 95% CI 0.05–0.65, *p* = 0.016, ≤ 14 years: aOR 0.55, 95% CI 0.13–1.66, *p* = 0.344). Underlying cardiovascular diseases (aOR 1.37, 95% CI 1.09–1.72, *p* = 0.007), diabetes mellitus (aOR 1.33, 95% CI 1.04–1.69, *p* = 0.023), and male gender (aOR 1.23, 95% CI 1.01–1.50, *p* = 0.040) were also significantly associated with a complicated clinical stage at SARS-CoV-2 detection.

**Table 2:** Associations between baseline characteristics and complicated clinical stage at day of positive SARS-CoV-2 detection

	Univariate model*		Mutivariable model*	
	OR (95% CI)	*p* value	aOR (95% CI)	*p* value
Age (years)				
≤ 14	0.51 (0.12–1.52)	0.279	0.55 (0.13–1.66)	0.344
15–25	0.21 (0.05–0.59)	**0.010**	0.23 (0.05–0.65)	**0.016**
26–45	ref.	ref.	ref.	ref.
46–65	2.07 (1.52–2.84)	** < 0.001**	1.73 (1.25–2.42)	**0.001**
66–85	2.74 (2.03–3.74)	** < 0.001**	1.93 (1.36–2.74)	** < 0.001**
> 85	3.29 (2.18–4.99)	** < 0.001**	2.38 (1.49–3.81)	** < 0.001**
Sex				
Female	ref.	ref.	ref.	ref.
Male	1.26 (1.05–1.51)	**0.013**	1.23 (1.01–1.50)	**0.040**
Body mass index (kg/m^2^)				
< 18.5	0.97 (0.46–1.95)	0.926	**	**
18.5–24.9	ref.	ref.	ref.	ref.
25–29.9	1.22 (0.93–1.62)	0.153	**	**
30–34.9	1.52 (1.07–2.16)	**0.019**	**	**
> 35	2.21 (1.43–3.43)	** < 0.001**	**	**
Smoking				
Active smoking	1.50 (1.02–2.18)	**0.037**	**	**
Former smoking	1.58 (1.10–2.27)	**0.013**	**	**
Nonsmoking	ref.	ref.	ref.	ref.
Comorbidities^1^				
Cardiovascular disease	2.00 (1.66–2.41)	** < 0.001**	1.37 (1.09–1.72)	**0.007**
Diabetes mellitus	1.76 (1.40–2.20)	** < 0.001**	1.33 (1.04–1.69)	**0.023**
Pulmonary disease	1.39 (1.08–1.78)	**0.009**	1.27 (0.98–1.64)	0.068
Hematological and/or oncological disease	1.00 (0.77–1.28)	0.977	***	***
Neurological disease	1.41 (1.12–1.76)	**0.003**	***	***
Kidney disease	1.54 (1.20–1.97)	** < 0.001**	***	***
Other comorbidities	0.89 (0.67–1.18)	0.438	***	***
Pre-existing medication at day of positive SARS-CoV-2 test^1^				
ACE inhibitors	1.45 (1.15–1.83)	**0.002**	***	***
AT-1-receptor antagonists	1.21 (0.95–1.54)	0.120	***	***
Statins	1.40 (1.09–1.80)	**0.009**	***	***
Ibuprofen	0.58 (0.33–0.97)	**0.047**	***	***
Pre-existing medication until three months before positive SARS-CoV-2 test^1^				
Any immunosuppressive medication	0.79 (0.57–1.09)	0.166	***	***

*n* = 134 Observations were excluded from multivariable regression model due to missingness. *ACE inhibitors* angiotensin-converting-enzyme inhibitor, *AT-1-receptor antagonists* angiotensin II type 1 (AT1) receptor antagonists, *Ref.* reference group. Other comorbidities—connective tissue disease, peptic ulcer disease, chronic liver disease, liver cirrhosis, organ transplantation, rheumatic disease, HIV/AIDS

*Results from a logistic regression model displayed with odds ratios (ORs) and 95% confidence intervals (CI); all variables were fitted simultaneously in the multivariable model

**Variable excluded from multivariable analysis due to multicollinearity

***Variable excluded from multivariable analysis due to model quality

^1^Reference group was not present/given

## Discussion

In this European study, we assessed the clinical characteristics of SARS-CoV-2-infected patients who required medical care and determined associating factors for complicated clinical stages at the time of SARS-CoV-2 detection (severity of COVID-19 at diagnosis). In comparison with previous reports, we identified predominant characteristics including male gender, and underlying cardiovascular diseases, diabetes mellitus, and pulmonary diseases [5, 7–10, 20, 21]. In addition, neurological diseases were predominant in our cohort.

In line with the published data, our observation confirms that inflammatory parameters are likely to be elevated at the time of SARS-CoV-2 detection [9, 22]. In our cohort, we only found an elevation of CRP > 29 mg/L in almost two-third of patients in a complicated clinical stage at time of COVID-19 diagnosis. However, our endpoint included pulmonary and extrapulmonary clinical findings. Patients without pneumonia were also defined as complicated clinical stage at time of COVID-19 diagnosis which could explain the less frequent increase in CRP. Further analysis on clinical course and outcome data might elucidate the potential prognostic significance of acute phase proteins such as ferritin and procalcitonin [4, 9, 23]. Body temperature at the time of SARS-CoV-2 detection was less frequently elevated in our cohort than observed in other studies assessing hospitalized patients [5, 7]. However, our cohort also included hospitalized patients whose admission was not related to COVID-19. Almost one-tenth of patients were asymptomatic. This finding demands further follow-up analyses in our cohort regarding clinical course and outcome. However, we detected elevated respiratory rates at days of positive SARS-CoV-2 testing which is adequate for a hospital setting [5, 20] and tends to be an important monitoring parameter.

As stated in other studies [21], over one-third of patients were diagnosed with COVID-19 in a complicated clinical stage. In line with other studies that examined risk factors for COVID-19 disease progression, we identified a significant univariate association between advanced age; male gender; adiposity; smoking; cardiovascular, pulmonary, and kidney diseases; and diabetes mellitus and complicated clinical stages at SARS-CoV-2 detection [5, 8, 9, 20, 24].

When comparing the observed significant univariate association of neurological diseases with complicated clinical stages at SARS-CoV-2 detection, the heterogeneity of the specific diseases defined as neurological diseases in this study should be considered. Furthermore, one must be cautious with the interpretation of the significant univariant factors such as pre-existing statin, ACE inhibitors, and ibuprofen intake on the day of SARS-CoV-2 detection. In the covariate adjusted model, these parameters were excluded due to decrease in model quality (increase of information loss). Additionally, other studies confirmed that pre-existing ACE inhibitors or ibuprofen intake are possibly not the factors causing severity of COVID-19 [16, 25–27]. However, there is a higher prevalence of cardiovascular diseases in patients with more severe COVID-19 illness [25].

Within a multivariable model, we found advanced age, male sex, underlying cardiovascular diseases, and diabetes mellitus as independent risk factors for complicated clinical disease at the time of diagnosis. Higher BMI and smoking as possible independent risk factors [9] were excluded due to multicollinearity. Further analysis would be necessary for incorporating dependencies of baseline characteristics to perform risk stratification.

This study analyzed associating factors for a complicated clinical stage at initial presentation (day of SARS-CoV-2 detection). The relation to severity of clinical course was not considered. Besides the clinical factors associated for a progressive disease more confounders might be important factors neglected in this study. Data on socioeconomics, insurance issues, access to health services, country specific testing capacities, testing policies or intrinsic motivation were not considered for this study. The mentioned factors could be correlated with a late diagnosis. Besides rapid COVID-19 progression, late diagnosis could be a reason for being in a complicated clinical stage at initial presentation.

Further limitations of our study include the limited number of patients considered in this study. Patients with a complicated clinical stage at SARS-CoV-2 detection could be underrepresented in this cohort, as more of these patients require long medical observation and patient’s outcome could more likely not be known at time of conduction of this analysis (inclusion criteria). The highest documentation rates were performed by university hospitals in larger cities; consequently, rural areas might be underrepresented. Most patients were documented in Germany, and results may, therefore, not be transferable to all regions of Europe. Furthermore, many hospitals in Germany had the capacity to monitor patients with asymptomatic or mild SARS-CoV-2 infections which would not be applicable to all European hospitals. Patients were mainly recruited from hospitals, and a transferability to outpatient settings is therefore limited. Comorbidities and medication intake were collected as binary categories. We did not incorporate data on the extent of underlying comorbidity nor regarding dose nor the duration of treatment. Comorbidities were additionally dichotomized; we did not consider associations of specific underlying diseases, as, for example, hypertension for cardiovascular diseases.

## Conclusions

To conclude, this comprehensive description identified characteristics of SARS-CoV-2-infected patients seeking medical care and determined associating factors for complicated clinical stages at diagnosis. The results indicated characteristics comparable to those observed in international published cohorts.

## Electronic supplementary material

Below is the link to the electronic supplementary material.

Supplementary file1 (DOCX 60 kb)
