# Supplementary material for: First results of the “Lean European Open Survey on SARS-CoV-2-Infected Patients (LEOSS)”
Source: Infection. 2020 Oct 1;49(1):63–73. doi: 10.1007/s15010-020-01499-0 (PMC7527665; doi:10.1007/s15010-020-01499-0)
Supplement: Supplementary file 1 — Supplementary file1 (DOCX 60 kb) [file 15010_2020_1499_MOESM1_ESM.docx]

**Supplements**

Supplements Table 1: Baseline characteristics of asymptomatic patients at day of positive SARS-CoV-2 detection

|  |  |
| --- | --- |
|  |  |
|  |  |
| **Cases relative to all patients (excluding missing information)** | 9.9% (161/1,629) |
| **Age (years)** |  |
| ≤ 14 | 1.2% (2/161) |
| 15 - 25 | 4.3% (7/161) |
| 26 – 45 | 15.5% (25/161) |
| 46 – 65 | 29.2% (47/161) |
| 66 – 85 | 40.4% (47/161) |
| >85 | 9.3% (15/161) |
| **Sex** |  |
| Female | 38.5% (62/161) |
| Male | 61.5% (99/161) |
| **Body-mass-index (kg/m²)** |  |
| < 18,5 | 4.6% (5/109) |
| 18,5 – 24,9 | 51.4% (56/109) |
| 25 – 29,9 | 28.4% (31/109) |
| 30 – 34,9 | 11.9% (13/109) |
| > 35 | 3.7% (4/109) |
| **Smoking** |  |
| Active smoking | 13.1% (11/84) |
| Former smoking | 16.7% (14/84) |
| Nonsmoking | 13.1% (11/84) |
| **Comorbidities** |  |
| Cardiovascular disease | 57.0% (90/158) |
| Diabetes mellitus | 23.6% (38/161) |
| Pulmonary disease | 12.1% (19/157) |
| Hematological and/or oncological disease | 22.0% (35/159) |
| Neurological disease | 33.1% (49/148) |
| Kidney disease | 21.4% (35/161) |
| Other comorbidities | 14.3% (22/154) |
| **Pre-existing medication at day of positive SARS-CoV-2 test** |  |
| ACE inhibitors | 15.8% (25/158) |
| AT-1-receptor antagonists | 18.9% (30/159) |
| Statins | 22.7% (32/141) |
| Ibuprofen | 3.2% (5/154) |
| **Pre-existing medication until three months before positive SARS-CoV-2 test** |  |
| Any immunosuppressive medication | 13.2% (19/144) |

ACE inhibitors (Angiotensin-converting-enzyme inhibitor). AT-1-receptor antagonists (angiotensin II type 1 (AT1) receptor antagonists). Other comorbidities (connective tissue disease, peptic ulcer disease, chronic liver disease, liver cirrhosis, organ transplantation, rheumatic disease, HIV/AIDS).

**Supplements Table 2**. Associations between baseline characteristics and complicated clinical stage at day of SARS-CoV-2 detection including variables with significant univariate association

|  |  | |  | |  |  |
| --- | --- | --- | --- | --- | --- | --- |
|  | Univariate model* | | | | Mutivariable model* | |
|  |  | OR (95% CI) | | p-value | aOR (95% CI) | p-value |
| **Age (years)** |  | |  | |  |  |
| ≤ 14 | 0.51 (0.12 – 1.52) | | 0.279 | | 0.31 (0.02 – 1.59) | 0.259 |
| 15 - 25 | 0.21 (0.05 – 0.59) | | **0.010** | | 0.31 (0.05 – 1.10) | 0.122 |
| 26 – 45 | ref. | | ref. | | ref. | ref. |
| 46 – 65 | 2.07 (1.52 – 2.84) | | **<0.001** | | 2.19 (1.44 – 3.40) | **<0.001** |
| 66 – 85 | 2.74 (2.03 – 3.74) | | **<0.001** | | 2.33 (1.50 – 3.71) | **<0.001** |
| >85 | 3.29 (2.18 – 4.99) | | **<0.001** | | 3.32 (1.85 – 6.01) | **<0.001** |
| **Sex** |  | |  | |  |  |
| Female | ref. | | ref. | | ref. | ref. |
| Male | 1.26 (1.05 – 1.51) | | **0.013** | | 1.21 (0.95 – 1.54) | 0.122 |
| **Body-mass-index (kg/m²)** |  | |  | |  |  |
| < 18,5 | 0.97 (0.46 – 1.95) | | 0.926 | | ** | ** |
| 18,5 – 24,9 | ref. | | ref. | | ref. | ref. |
| 25 – 29,9 | 1.22 (0.93 – 1.62) | | 0.153 | | ** | ** |
| 30 – 34,9 | 1.52 (1.07 – 2.16) | | **0.019** | | ** | ** |
| > 35 | 2.21 (1.43 – 3.43) | | **<0.001** | | ** | ** |
| **Smoking** |  | |  | |  |  |
| Active smoking | 1.50 (1.02 – 2.18) | | **0.037** | | ** | ** |
| Former smoking | 1.58 (1.10 – 2.27) | | **0.013** | | ** | ** |
| Nonsmoking | ref. | | ref. | | ref. | ref. |
| **Comorbidities^1^** |  | |  | |  |  |
| Cardiovascular disease | 2.00 (1.66 – 2.41) | | **<0.001** | | 1.20 (0.89 – 1.64) | 0.234 |
| Diabetes mellitus | 1.76 (1.40 – 2.20) | | **<0.001** | | 1.29 (0.96 – 1.73) | 0.094 |
| Pulmonary disease | 1.39 (1.08 – 1.78) | | **0.009** | | 1.23 (0.88 – 1.69) | 0.216 |
| Hematological and/or oncological disease | 1.00 (0.77 – 1.28) | | 0.977 | | *** | *** |
| Neurological disease | 1.41 (1.12 – 1.76) | | **0.003** | | 0.90 (0.66 – 1.21) | 0.485 |
| Kidney disease | 1.54 (1.20 – 1.97) | | **<0.001** | | 1.04 (0.75 – 1.44) | 0.817 |
| Other comorbidities | 0.89 (0.67 – 1.18) | | 0.438 | | *** | *** |
| **Pre-existing medication at day of positive SARS-CoV-2 test^1^** |  | |  | |  |  |
| ACE inhibitors | 1.45 (1.15 – 1.83) | | **0.002** | | 1.10 (0.81 – 1.49) | 0.534 |
| AT-1-receptor antagonists | 1.21 (0.95 – 1.54) | | 0.120 | | *** | *** |
| Statins | 1.40 (1.09 – 1.80) | | **0.009** | | 0.98 (0.73 – 1.32) | 0.918 |
| Ibuprofen | 0.58 (0.33 – 0.97) | | 0.047 | | *** | *** |
| **Pre-existing medication until three months before positive SARS-CoV-2 test^1^** |  | |  | |  |  |
| Any immunosuppressive medication | 0.79 (0.57 – 1.09 | | 0.166 | | *** | *** |

*n= 799 observations were excluded from multivariable regression model due to missingness. ACE inhibitors (Angiotensin-converting-enzyme inhibitor). AT-1-receptor antagonists (angiotensin II type 1 (AT1) receptor antagonists). Ref. (reference group). Other comorbidities (connective tissue disease, peptic ulcer disease, chronic liver disease, liver cirrhosis, organ transplantation, rheumatic disease, HIV/AIDS).*

**Results from a logistic regression model displayed with odds ratios (ORs) and 95% confidence intervals (CI); all variables were fitted simultaneously in the multivariable model.*

***Variable excluded from multivariable analysis due to multicollinearity.*

****Variable excluded from multivariable analysis.*

*^1^reference group was not present/given*
